# Whole-genome sequencing of eight goat populations for the detection of selection signatures underlying production and adaptive traits

**DOI:** 10.1038/srep38932

**Published:** 2016-12-12

**Authors:** Xiaolong Wang, Jing Liu, Guangxian Zhou, Jiazhong Guo, Hailong Yan, Yiyuan Niu, Yan Li, Chao Yuan, Rongqing Geng, Xianyong Lan, Xiaopeng An, Xingui Tian, Huangkai Zhou, Jiuzhou Song, Yu Jiang, Yulin Chen

**Affiliations:** 1 College of Animal Science and Technology, Northwest A&F University, Yangling, 712100, China; 2 College of Animal Science and Technology, Sichuan Agricultural University, Ya’an, 625000, China; 3 College of Life Science, Yulin University, Yulin, 719000, China; 4 Lanzhou Institute of Husbandry and Pharmaceutical Sciences of CAAS, Lanzhou 730050, China; 5 College of Pharmacy, Yancheng Teachers University, Yancheng, 224051, China; 6 Guizhou University, Guiyang, 550000, China; 7 Guangzhou Gene de-novo Biotechnology Co. Ltd. Guangzhou, 510000, China; 8 Department of Animal and Avian Sciences, University of Maryland, College Park, Maryland 20742,USA

## Abstract

The goat (*Capra hircus*) is one of the first farm animals that have undergone domestication and extensive natural and artificial selection by adapting to various environments, which in turn has resulted in its high level of phenotypic diversity. Here, we generated medium-coverage (9–13×) sequences from eight domesticated goat breeds, representing morphologically or geographically specific populations, to identify genomic regions representing selection signatures. We discovered ~10 million single nucleotide polymorphisms (SNPs) for each breed. By combining two approaches, ZH_p_ and di values, we identified 22 genomic regions that may have contributed to the phenotypes in coat color patterns, body size, cashmere traits, as well as high altitude adaptation in goat populations. Candidate genes underlying strong selection signatures including coloration (*ASIP*, *KITLG*, *HTT*, *GNA11*, and *OSTM1*), body size (*TBX15*, *DGCR8*, *CDC25A*, and *RDH16*), cashmere traits (*LHX2*, *FGF9*, and *WNT2*), and hypoxia adaptation (*CDK2*, *SOCS2*, *NOXA1*, and *ENPEP*) were identified. We also identified candidate functional SNPs within selected genes that may be important for each trait. Our results demonstrated the potential of using sequence data in identifying genomic regions that are responsible for agriculturally significant phenotypes in goats, which in turn can be used in the selection of goat breeds for environmental adaptation and domestication.

The goat (*Capra hircus*) is believed to be one of the first livestock species that underwent domestication approximately 10,000 years ago^1^,^2^, and therefore has been a witness to the historical progress of human civilization. A variety of natural or artificial factors (e.g., environmental changes, human migration, and socioeconomic influences) have shaped the phenotypic diversity of goats, leading to 557 registered goat breeds worldwide (FAO)^3^. Since the Neolithic age, goats have played economically important roles by providing various products (e.g., fiber, milk, meat, and hide) to the human population.

Artificial selection during domestication and production-oriented breeding has greatly shaped the level of genomic variability in goats. The genome of goats, which have diverse production potentials and extensive adaptation to diverse environments, provides a unique opportunity for identifying signatures associated with selection. Array- or sequencing-based detection of signatures during the selection process has been described in cattle^4^,^5^,^6^, chicken^7^, pigs^8^,^9^,^10^, sheep^11^,^12^, and recently in goats^13^,^14^,^15^. By using whole-genome resequencing (WGS) data, Benjelloun *et al*. identified positive selection sweeps of three indigenous goat populations in Morocco^14^, whereas Dong *et al*. described selection signals during goat domestication by analyzing the sequences of domestic goats and its wild progenitor, *bezoar*^15^. However, investigations on selection signatures with respect to selection purposes and breed formation of goats are limited. WGS using a group of DNA samples (Pool-seq) from the same individuals permits molecular biologists to simultaneously examine sequence divergence among populations, morphs, or breeds for hundreds of genes and gene families in a cost-efficient manner, especially for well-studied species with large genome sizes^7^,^16^.

The aim of this study was to detect evidence of signatures of recent selection among goats for different selection objectives. To do this, we investigated selection signatures using pooling sequencing of eight distinct goat populations (Supplementary Table S1), including a black coated breed (Taihang Black), a highland breed (Tibetan goat), two cashmere goat breeds (Inner Mongolia Cashmere and Shaanbei Cashmere) a breed for mohair (Angora), a dairy goat population (Saanen), a meat goat breed (Boer), and a mini goat breed with small body size (Guizhou Small) (Fig. 1a and b). By performing combined calculations for the ZH_p_ and di values of all autosome SNPs, we observed a small number of strong selection signatures near known artificial selective genes in other animals. Our findings can be used to better understand genomic signatures under selection, as well as shed some light on genomic regions that harbor genes controlling production or adaptive traits in goats.

## Results and Discussion

### Genome resequencing of eight goat breeds

**Figure 1:**
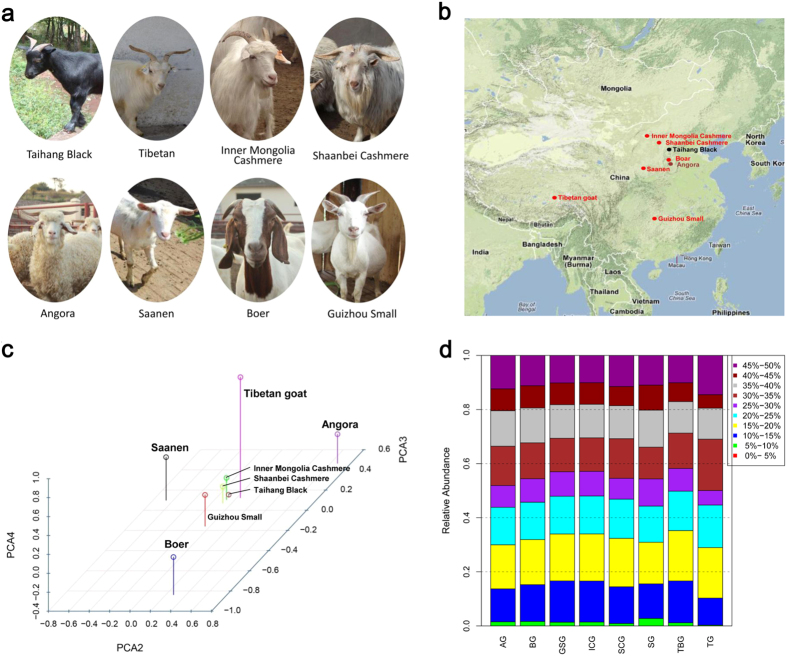
Summary of eight goat breeds. **(a)** The eight goat breeds included in this study (Photographs were taken by Xiaolong Wang and Xingui Tian). (**b)** Geographic map indicating the distribution of the goats sampled in this study. Each point represents the location of sampling. The map was generated using the ‘ggmap’ package in R (version 3.1.0)^60^. **(c)** Principal components analysis (PCA) of eight goat breeds using components PC2, PC3, and PC4. **(d)** A schematic representation of MAF plotted as a function of distance for each goat population. Angora (AG), Boer (BG), Guizhou Small (GSG), Inner Mongolia Cashmere (ICG), Shaanbei Cashmere (SCG), Saanen (SG), Taihang Black (TBG), and Tibetan (TG).

Eight genetically diverse domestic goat breeds with different production purposes were used to systematically investigate the selection signatures in goats (Fig. 1b, Supplementary Table S1). WGS was performed on an Illumina HiSeq 2000 platform by using the pooled DNA from each breed. Genome sequencing yielded a total of 203 Gb raw data, and produced 219 to 350 million sequence reads per breed (Table 1). Over 97.5% of the generated sequence reads mapped to approximately 93.73% (93.19–94.07%) of the newly annotated goat reference genome (CHIR_2.0), indicating that high quality sequences were obtained. Our efforts yielded an average sequence coverage of 10.5× per breed, within a range of 9- to 13-fold. Single-nucleotide polymorphisms (SNPs) varied from 8–9 million for each population (Table 2).

Principal components analysis (PCA) was performed to examine the genetic separation of eight goat breeds (Fig. 1c, Supplementary Fig. S1). PCA clearly classified the three introduced breeds (Saanen, Boer, and Angola) and other Chinese indigenous goat breeds. Analysis of breeding history of these five Chinese indigenous breeds, confirmed the results of the PCA; for example, two cashmere goat breeds (Inner Mongolia Cashmere and Shaanbei Cashmere) were clustered together, and were closely related to Taihang Black, both genetically and geographically. The cluster results were in agreement with the findings of a previous study on the genetic diversity of goat breeds in China^17^.

### Identification of coding SNPs and short insertions/deletions

More than nine million SNPs for each breed that confidently remained after filtering were used in the subsequent analyses. Around 74–88% of all the SNPs were heterozygous, and Shaanbei Cashmere and Guizhou Small have the largest proportion of heterozygous SNPs, indicating these two underwent recent intensive selection. Only a few SNPs (~0.5%) were located within coding regions. The non-synonymous and synonymous variants were also identified in the goat genome (Table 2), and there were more synonymous variants than non-synonymous substitutions. The proportion of non-synonymous SNPs in each breed was stable (~43.5%) (Supplementary Fig. S2), which is close to the proportion of non-synonymous SNPs in cattle^18^. We also identified a large number of SNPs with large effects (premature stop codons, start codon to non-start codon, stop codon to non-stop, and splice site) across the goat genome (Supplementary Fig. S3).

The distribution of minor allelic frequency (MAF) with 10 continued classes from 0–0.05 to 0.45–0.50 for each breed was observed (Fig. 1d). The largest group of the SNPs had a MAF within the range of 0.15–0.20 (16–18%), whereas the proportion of rare alleles (MAF < 0.05) only accounted for <0.01% of the total SNPs, because most of the rare alleles were removed during the SNP calling process.

### Identification of selective loci and candidate genes

To detect genomic regions related to selection in domesticated animals, several statistical methods have been developed such as overall low heterozygosity^7^,^9^, genetic diversity patterns^19^, haplotype homozygosity^20^, and integrated haplotype score (|iHS|)^21^. To detect putative selective loci in the present study, we first calculated the pooled heterozygosity (H_p_) and its Z transformations, ZH_p_, in sliding 150-kb windows along the autosomes as previously described^7^,^9^. We further selected the top 1% of the SNPs with the highest di values as differentiated genomic regions among breeds^22^. Putative genomic regions that overlapped between these two approaches were defined as candidate selective loci.

#### Coat Color

**Figure 2:**
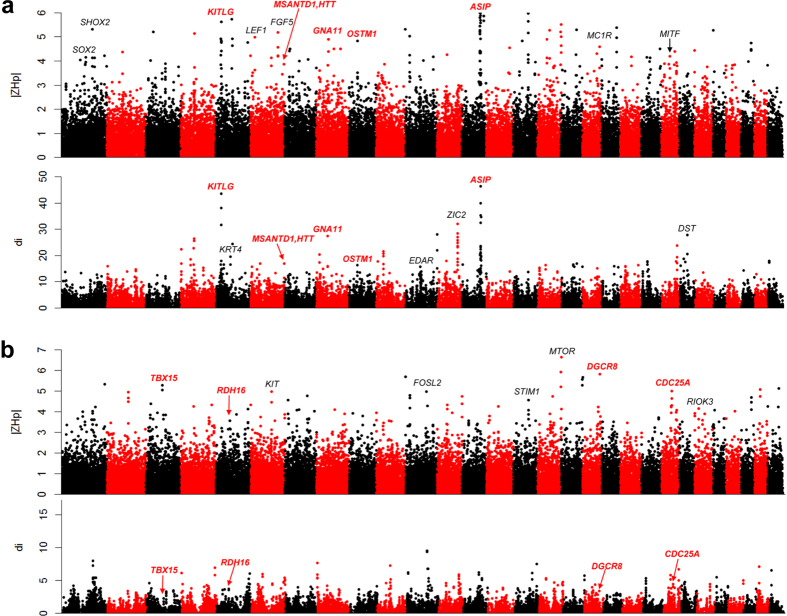
Overview of selective sweeps in the Taihang Black and Guizhou Samll breeds plotted by ZH_p_ and di values. **(a)** Taihang Black goat breed. **(b)** Guizhou Samll goat. Functional genes are highlighted, red and bold characters represent overlapping genes that were generated by using the two methods. Absolute values of ZH_p_ were used for plotting.

By analyzing the heterozygosity of Taihang Black, 48 distinct loci specific for the Taihang Black breed were identified (ZH_p _≤ −4), including well known coat color genes *ASIP*, *MC1R, MITF*, and *KITLG* (Fig. 2a, Supplementary Dataset S1 and S2). MC1R plays key roles in the regulation of eumelanin (black/brown) and phaeomelanin (red/yellow) synthesis in mammalian melanocytes. Mutations in the *MC1R* gene have been associated with coat colors variation in pigs^23^,^24^, cattle^25^, and goats^26^. As an antagonist to *MC1R* to stimulate pheomelanin synthesis, *ASIP* has been implicated as a strong candidate gene that controls coat color patterns in goats and sheep^27^,^28^,^29^. MITF is a key regulator of melanocyte development and is associated with various coat patterns in mammals^30^. *KITLG*, which encodes for the ligand of c-Kit, plays a role in the melanocyte production pathway, and variations in the *KITLG* locus have been associated with coat color patterns in pigs^31^, and cattle^32^. In addition, a total of 54 genomic regions were found within the top 1% distribution (di > 10.82), and a list of candidate genes was generated. Among these, 29 genes were listed by the European Society for Pigment Cell Research (http://www.espcr.org/micemut) (only 150 genes were well annotated in autosomes of goat genome), suggesting that these genes might also play important roles in coat color formation in domestic goats.

Six loci overlapped between the genetic regions with the lowest ZH_p_ values and highest di values. Five overlapped regions contained strongest candidate genes (*ASIP*, *KITLG*, *MSANTD1*, *HTT*, *GNA11*, and *DST*) that were specific to Taihang black (Table 3). A locus encompassing the *MSANTD1* and *HTT* genes was recently identified as the strongest selective sweep in European black goat populations^14^, thereby highlighting the importance of this locus in the determination of black coat color in goats. Given that the *HTT* gene plays an important role in nerve cells (neurons) in the brain and takes part in pigments associated with aging and diseases, such as Huntington disease^33^, thus it is likely that *HTT* is the candidate gene in this locus that is responsible for coat color. *GNA11* and *OSTM1* were listed as coat color genes in mice (http://www.espcr.org/micemut). These results further indicate the reliability to identify strong selective genes using this approach.

#### Body size

The Guizhou Small goats originated from the remote mountain area of the Guizhou Province in southwest China. To maintain its small physical figure and meat taste, intercrosses are often made and the population size of the Guizhou Small has become smaller^34^. Compared to the body weight of larger meat goat breeds, e.g. Boer, which could weigh over 100 kg, the average body weight of the Guizhou Small is as low as ~20 kg in females and ~25 kg in males. Therefore, body size trait of Guizhou Small could be beneficial in increasing carcass weight, and should be considered in meat goat breeding programs.

A total of 49 regions related to Guizhou Small breeds were mapped with a ZH_p_ value of <−4. Strong selection signals including known genes *FOSL2*, *DGCR8*, *MTOR*, and *TBX15* were localized. We discovered 56 regions that were within the top 1% distribution of the di values. Only four functional genes (*TBX15*, *DGCR8*, *CDC25A*, and *RDH16*) within four loci overlapped showed low ZH_p_ values and high divergence (Fig. 2b, Supplementary Dataset S1 & S2). *TBX15* controls the number of mesenchymal precursor cells and chondrocytes, and is essential to skeletal development^35^. Osteoclast-specific deletion of DGCR8 results in impaired osteoclastic development and bone resorbing activity, indicating that the *DGCR8* gene is essential for bone development^36^. *CDC25A* plays important roles in G1 quiescence and myogenic differentiation of myoblasts in mice^37^. The *RDH16* gene is involved in energy and metabolism processes in adipose tissues in pigs^38^,^39^, and rats^40^.

#### Cashmere traits

In mammals, coat hair acts as a protective material against environmental changes. Unlike other mammals, cashmere-producing goats have a double coat consisting of the outer coarse hair produced by primary hair follicles (PHF) and the inner fine coat (cashmere) produced by secondary hair follicles (SHF). In contrast, the coat hair of the Angora goat exclusively produces a fleece of fibers named mohair, which is generated by SHF with limited proportion of guard hair from PHF^41^. In the case of cashmere fibers, selection for an optimal fiber diameter with an increased fiber length is the long-term goal of cashmere goat breeding programs. Although earlier studies have assessed only a few candidate genes [e.g., *POU1F1*^42^, and *PRL*^43^] that associated with cashmere traits (cashmere yield, cashmere diameter and length), the genetic determinants controlling cashmere traits in goats have remained largely elusive at a genome level.

**Figure 3:**
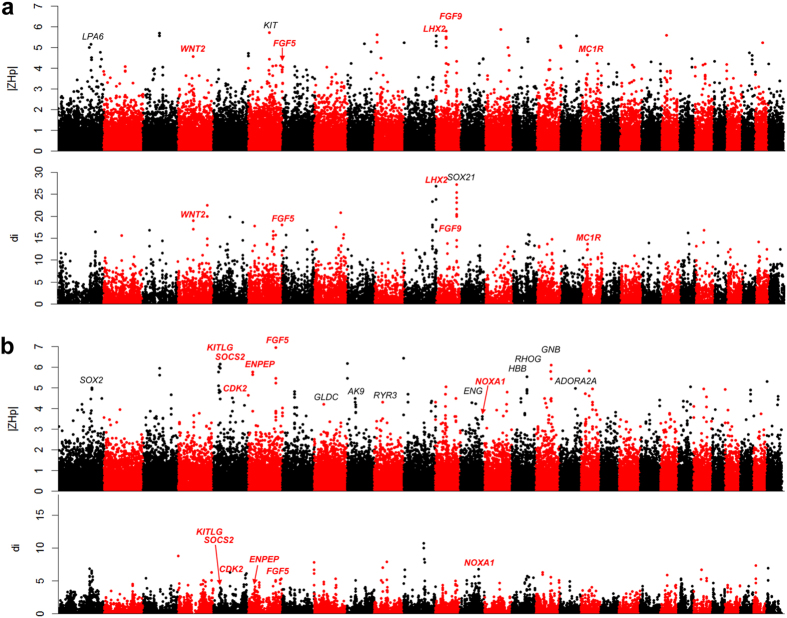
Overview of selective sweeps in the Inner Mongolian Cashmere and Tibetan goats plotted by ZH_p_ and di values. (**a**) Inner Mongolian Cashmere breed. (**b**) Tibetan goats breed.

By analyzing the sequence heterozygosity and divergence of a well-known cashmere goat breed, Inner Mongolia Cashmere, with other goat breeds, 40 and 37 genomic regions were implicated to be cashmere goat-specific regions using ZH_p_ and di methods, respectively (Fig. 3a, Supplementary Dataset S1 & S2). We merged the regions that were generated by these two approaches to identify the strongest signature of selection. Five regions encompassing the *LHX2*, *FGF9*, *WNT2*, *MC1R*, and *FGF5* were detected. *LHX2*, a LIM homeobox gene, regulates the generation and regeneration of hair^44^. We have previously shown that the cyclic expression of *LHX2* is involved in the development of SHF in cashmere goats^45^. *FGF9* is able to promote hair follicle regeneration after wounding^46^, and the Wnt-related genes *WNT2* is a key mediator and regulator of Wnt signaling, and is involved in hair follicle initiation^47^. These two genes may explain the cyclic growth of cashmere fibers in cashmere goats. Furthermore, the coat color gene *MC1R* controls white coat color and *FGF5* that regulates hair length were also mapped, consistent with the selection purposes (such as white and longer fibers) of cashmere goats.

#### High altitude adaptation

The Tibetan goat, together with the yak and Tibetan sheep, are the three major livestock species that serve as sources of meat and fibers for Tibetan inhabitants. Endemic to the Tibetan Plateau, the Tibetan goats are well adapted to high altitudes, often inhabiting open alpine and cold steppe environments located between 4,000 and 5,500 m elevation.

In the genome of the Tibetan goat, 49 loci were regarded as selected regions for adaptation to highland altitude environment based on the calculated ZH_p_ values (Fig. 3b, Supplementary Dataset S1 & S2). Several genes within these regions were previously implicated in the adaptation of Tibetan dwellers, including *HMOX2* and *HBB* in Tibetans^48^, *AK9* in Tibetan chickens^49^, *GLDC* and *RHOG* in Tibetan pigs^10^, *ATP12A*, *PIK3C2A, ADORA2A*, and *ENG* in Tibetan antelope^50^, *GNB1* in Tibetan dogs^51^. In addition, a total of 53 regions were within the top 1% of the distribution (di > 12.79), which included highland adaptation related genes such as *ANGPTL4* in Tibetans^48^*, ENO3* and *KIF1C* in Tibetan dogs^51^, and *PKLR* in Tibetan antelope^50^. We overlapped the genomic regions generated by these two approaches and identified seven regions that showed the strongest signature of selection by displaying both high di and low ZH_p_ values. Six genes (*CDK2*, *SOCS2*, *NOXA1*, *ENPEP*, *KITLG*, and *FGF5*) within these seven regions have plausible biological functions that are associated with high altitude adaptation or breed features. *CDK2* is a selected gene in the Tibetan mastiff^52^, and is involved in hypoxia-induced apoptosis in cardiomyocytes^53^. *SOCS2* is implicated as a selective gene in Tibetan sheep^54^. NOXA1 is the activator of NOX1, which is associated with HIF-1 response under intermittent hypoxia conditions^55^. *ENPEP* is a candidate gene for high-altitude adaption in Andeans^56^. Moreover, *FGF5*, a key regulator that controls hair length in mammals, was also identified and may explain the longer hair of Tibetan goats as an adaptation of cold environments of highlands. *KITLG,* a coat color gene, may be associated with white color in Tibetan goats.

**Figure 4:**
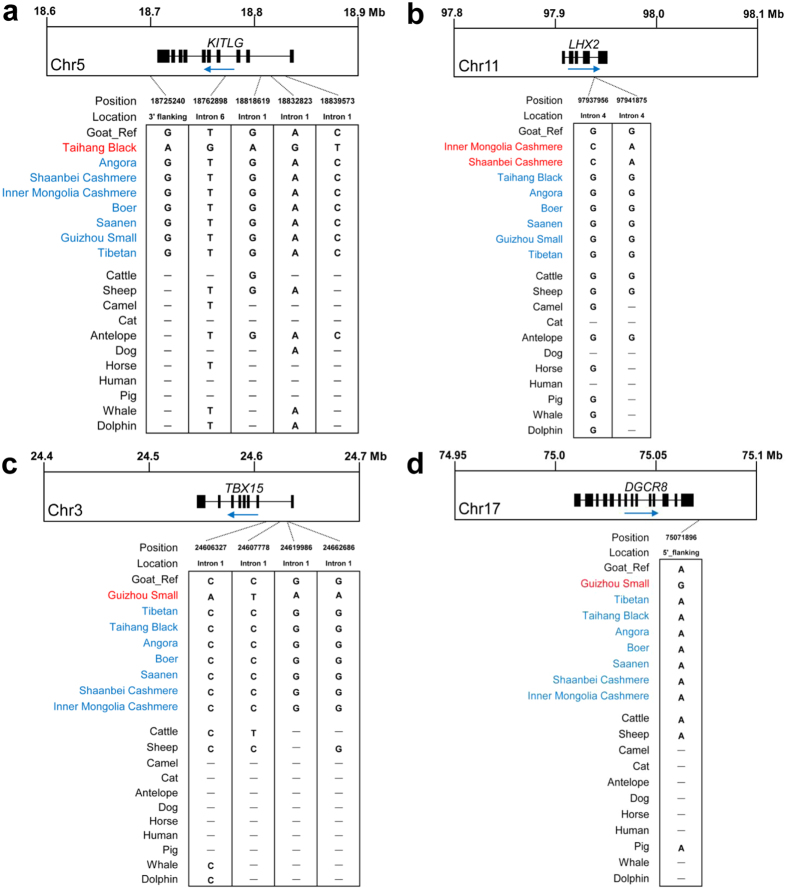
The conservation analyses identified candidate functional mutations that are specific to goat breeds. Breed-specific SNPs within or close to the key selected genes *KITLG* **(a)**, *LHX2* **(b)**, *TBX15* **(c)**, and *DGCR8* **(d)** were first screened, and the SNPs localized to evolutionary conserved sites were remained. The location and position of the candidate SNPs are indicated.

We further examined the functional importance of SNPs that were within seven representative selected genes (*KITLG*, *ASIP*, *LHX2*, *TBX15*, *DGCR8*, *CDK2*, and *SOCS2*), breed-specific SNPs among four genes (*KITLG*, *LHX2*, *TBX15*, and *DGCR8*) were localized to evolutionary conserved regions in mammals (Fig. 4a–d), suggesting that these SNPs might be functional. Consistent with a previous finding in rabbit domestication^57^, none of these conserved SNPs in these four genes were located within coding regions that lead to amino acid exchanges, thereby indicating that the genetic basis of goat production and adaptive traits are complex, and are rather regulatory variants.

Taken together, we conducted a comprehensive study to identify selection signatures in goats based on resequencing data. A total of 22 strong candidate regions with respect to distinct breeds were identified, which comprised genes involved in coat color patterns, body size, cashmere selection-specific phenotypes, as well as adaptation to low-oxygen environments in the highlands (Supplementary Table S2). Because no systematic mapping studies in goats at a genome-wide scale are currently available, the genes we highlighted may be regarded as the major candidate genes that are involved in shaping the particular characteristics of goat populations. For instance, we confirmed the importance of the *HTT* locus as a strong signal in the determination of black coat color in goats. Similarly, fine-scale mapping of selection signatures in goats may also facilitate in the interpretation and establishment of the molecular basis of economically important goat traits. For example, the availability of the Illumina goat SNP50 array^11^,^58^, as well as the rapid decrease in sequencing cost permit the identification of candidate economically important traits in goats. Our findings will facilitate the genetic dissection of phenotypic variation and aid in the future genetic improvement of production and adaptive traits in goats.

## Materials and Methods

### Ethics statement

The sampling procedures were compliance with the “Guidelines on Ethical Treatment of Experimental Animals” (2006) No. 398 established by the Ministry of Science and Technology, China. The sampling procedures in the present study had received prior approval from the Experimental Animal Manage Committee of Northwest A&F University (Approval ID: 2012ZX08008–002).

### Animals and whole genome sequencing

Over 20 animals from each breed were combined into a pool for high-throughput resequencing (Supplementary Table S1). DNA was extracted from whole blood samples by using the Qiagen DNeasy Blood and Tissue Kit (Qiagen, Hilden, Germany) and was used in the generation of paired-end libraries by using the Genomic DNA Sample Preparation Kit (Illumina, San Diego, CA). Briefly, 5 μg of DNA were sheared with a nebulizer and after end repair, A-tailing, and ligation of paired-end adapters, the library was size-selected on an agarose gel (300 bp) and amplified using 10 PCR cycles. Cluster amplification was performed using the Illumina Paired-End Cluster Generation Kit v2. Sequences were generated with the Illumina Sequencing Kit v3 and the Illumina Genome AnalyzerIIx System at Novogene (http://www.novogene.com). Image analysis was performed with Firecrest, Bustard, and Gerald modules of the Illumina pipeline v. 1.4. In total, 5 paired-end lanes were sequenced, which produced 80 mio PE fragments (160 mio individual reads), with an average read length of 74 bp.

### Reads alignment and variations calling

Reads were aligned to the goat reference genome CHIR_2.0 (http://www.ncbi.nlm.nih.gov/assembly/GCA_000317765.2/)^13^ using BWA (0.6.2-r126 version) followed by duplicate removal using Picard-Tools-1.55 (http://broadinstitute.github.io/picard/). The Genome Analysis Toolkit (GATK-2.6)^59^ was used to perform local realignment around existing indels and base quality score recalibration. Variant detection was performed using the GATK Unified Genotyper. To filter SNPs for flowing analysis, at least three reads with different start sites supporting the non-reference allele had to be present.

### Detection of selective loci

To identify regions that were likely to be or have been under selection, the “Z transformed heterozygosity” (ZH_p_) approach was used, as previously described^7^,^9^. In brief, in an overlapping sliding window, *H*_*p*_ was calculated as follows: 
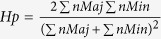
 is the sum of major allele frequencies, and Σ*nMin* is the sum of MAF within each window. Individual *H*_*p*_ values were Z transformed as follows: 
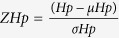
, where *μH*_*p*_ is the overall average heterozygosity, and *σH*_*p*_ is the standard deviation for all windows within each group. We calculated the ZH_p_ value in sliding 150-kb windows along the autosomes from sequence reads corresponding to the most and least frequently observed alleles at all SNP positions as previously described^7^,^9^. Because sex chromosomes and autosomes are subjected to different selective pressures and have different effective population sizes, we decided to calculate the ZH_p_ for autosomes of specific breeds only. Genetic differentiation between every pairwise comparison was measured by means of the fixation index (F_ST_) and *di* values were calculated as described by Akey *et al*.^22^ to evaluate population differentiation among specific breeds. Putatively selected loci were defined as genetic regions in overlapped windows with extremely low ZH_p_ values (<−4) and extremely high di values (top 1% level).

### Bioinformatics analysis of breed specific SNPs

Seven genes representing breed-specific selection signatures in Taihang Black (*KITLG* and *ASIP*), Guizhou Small (*TBX15* and *DGCR8*), cashmere breeds (*LHX2*), and Taibetan (*CDK2*, and *SOCS2*) were chosen for further analysis. We specifically focused on SNPs within genes and 1000-bp upstream and downstream flanking regions. We defined the breed-specific SNPs that differed from the goat reference sequence (CHI2.0) and other seven goat breeds used in the present study, and were localized to evolutionary conserved regions among mammal species.

## Additional Information

**How to cite this article:** Wang, X. *et al*. Whole-genome sequencing of eight goat populations for the detection of selection signatures underlying production and adaptive traits. *Sci. Rep.* **6**, 38932; doi: 10.1038/srep38932 (2016).

**Publisher's note:** Springer Nature remains neutral with regard to jurisdictional claims in published maps and institutional affiliations.

## Supplementary Material

Supplementary File

Supplementary Dataset 1

Supplementary Dataset 2

## Figures and Tables

**Table 1:** Results of Illumina sequencing and assembly.

Breed	Raw data (G)	Clean data (G)	Reads number (M)	Reads for alignment (%)	Sequence coverage
TBG	22.43	22.02	224.37	220.13 (98.11)	9.35×
TG	35.01	34.13	350.10	341.46 (97.53)	13.95×
ICG	23.30	22.86	233.03	228.62 (98.10)	9.67×
SCG	21.90	21.43	219.07	214.28 (97.81)	9.13×
AG	25.90	25.44	259.06	254.47 (98.23)	10.65×
SG	26.55	26.00	265.58	259.98 (97.89)	10.86×
BG	24.08	23.59	240.37	236.02 (98.20)	9.95×
GSG	24.17	23.72	241.61	237.34 (98.23)	10.0×

**Table 2:** Summary and annotation of SNPs and indels in the goat genome.

Breed	Number of animals	#SNPs	SNPs		Coding SNPs	
			Homo.	Hete. (%)	Non- syn. (%)	Syn.
SCG	29	10519612	1226326	9293286 (88.3)	26845 (43.4)	35010
TG	25	9469142	1659401	7809741 (82.5)	23720 (43.5)	30802
ICG	28	10282737	1332165	8950572 (87.0)	25732 (43.5)	33381
TBG	21	9896309	1508571	8387738 (84.8)	25107 (43.5)	32613
AG	20	9121167	2329265	6791902 (74.5)	24042 (43.9)	30775
SG	21	9813582	2112018	7701564 (78.5)	25104 (43.5)	32631
BG	22	9735835	1961090	7774745 (79.9)	25229 (43.7)	32473
GSG	21	9866776	1151745	8715031 (88.3)	24917 (43.7)	32136

**Table 3:** Overlapped genes that identified by both ZH_p_ and di for different goat breeds.

Candidate gene	Chr	Annotation	ZH_p_	di
Taihang Black				
* ASIP*	13	agouti signaling protein	−5.60	55.46
* KITLG*	5	KIT ligand	−5.83	43.56
* MSANTD1*, *HTT*	6	Myb/SANT DNA binding domain containing 1, huntingtin	−4.96	16.85
* GNA11*	7	G protein subunit alpha 11	−5.30	12.87
* OSTM1*	9	G protein subunit alpha 11	−4.94	16.35
Guizhou Small				
* TBX15*	3	T-box 15	−5.04	24.67
* DGCR8*	17	DGCR8 Microprocessor Complex Subunit	−6.06	32.60
* CDC25A*	22	cell division cycle 25A	−4.99	37.99
* RDH16*	5	retinol dehydrogenase 16	−6.06	37.14
Inner Mongolian Cashmere				
* LHX2*	11	LIM homeobox 2	−4.98	23.34
* FGF9*	12	fibroblast growth factor 9	−4.93	13.80
* WNT2*	4	Wnt family member 2	−4.29	19.04
* MC1R*	18	melanocortin 1 receptor	−5.52	13.73
* FGF5*	6	fibroblast growth factor 5	−6.12	15.68
Tibetan goat				
* CDK2*	5	cyclin dependent kinase 2	−6.45	39.75
* SOCS2*	5	suppressor of cytokine signaling 2	−5.25	33.18
* NOXA1*	11	NADPH oxidase activator 1	−4.53	22.83
* ENPEP*	6	glutamyl aminopeptidase	−5.65	17.41
* KITLG*	5	KIT ligand	−5.82	15.06
* FGF5*	6	fibroblast growth factor 5	−6.29	16.77
