# Supplementary material for: Whole-genome sequencing of eight goat populations for the detection of selection signatures underlying production and adaptive traits
Source: Sci Rep. 2016 Dec 12;6:38932. doi: 10.1038/srep38932 (PMC5150979; doi:10.1038/srep38932)
Supplement: Supplementary File [file srep38932-s1.pdf]

Supplementary Information

Whole-genome sequencing of eight goat populations for the detection of selection signatures underlying production and adaptive traits

Xiaolong Wang[1 §], Jing Liu[1 §], Guangxian Zhou[1], Jiazhong Guo[2], Hailong Yan[1,3], Yiyuan Niu[1], Yan Li[1], Chao Yuan[4], Rongqing Geng[1,5], Xianyong Lan[1], Xiaopeng An[1], Xingui Tian[6], Huangkai Zhou[7], Jiuzhou Song[8], Yu Jiang[1], Yulin Chen[1*]

[1]College of Animal Science and Technology, Northwest A&F University, Yangling, 712100 China.
[2]College of Animal Science and Technology, Sichuan Agricultural University, Ya'an, 625000 China.
[3]College of Life Science, Yulin University, Yulin, 719000 China.
[4]Lanzhou Institute of Husbandry and Pharmaceutical Sciences of CAAS，Lanzhou 730050 China.
[5]College of Pharmacy, Yancheng Teachers University, Yancheng, 224051 China.
[6]Guizhou University, Guiyang, 550000 China.
[7]Guangzhou Gene de-novo Biotechnology Co. Ltd. Guangzhou, 510000 China.
[8]Department of Animal and Avian Sciences, University of Maryland, College Park, Maryland 20742 USA.

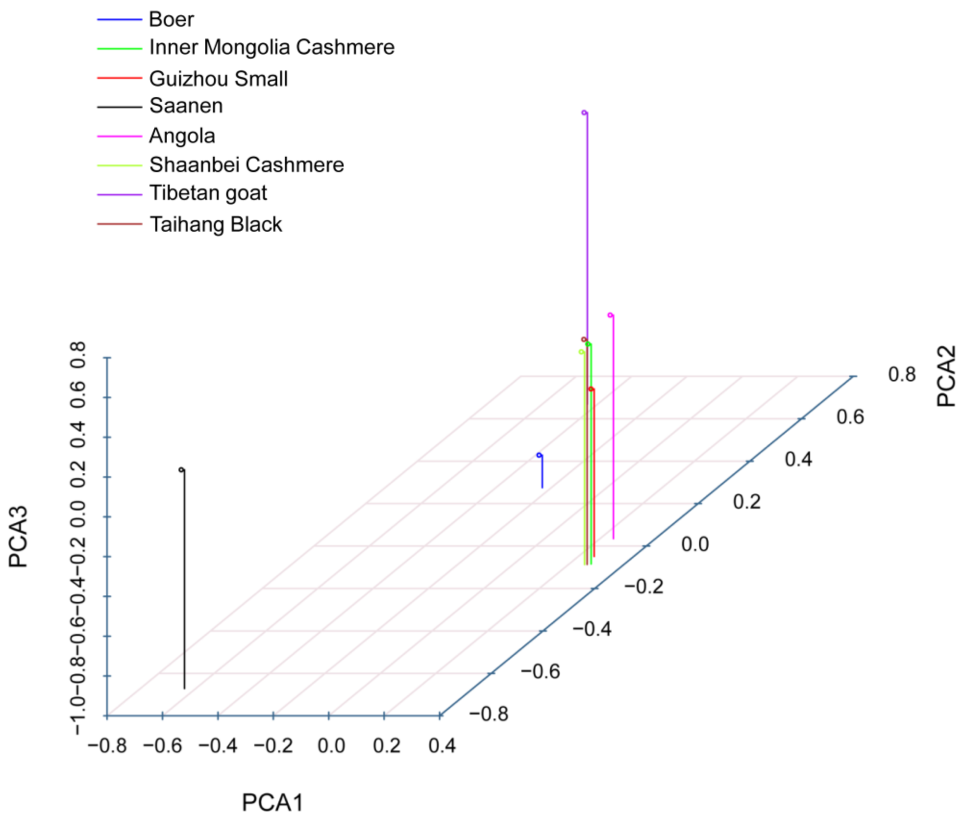

Supplementary Fig. S1 Principal components analysis (PCA) of eight goat breeds using components PC1, PC2, and PC3.

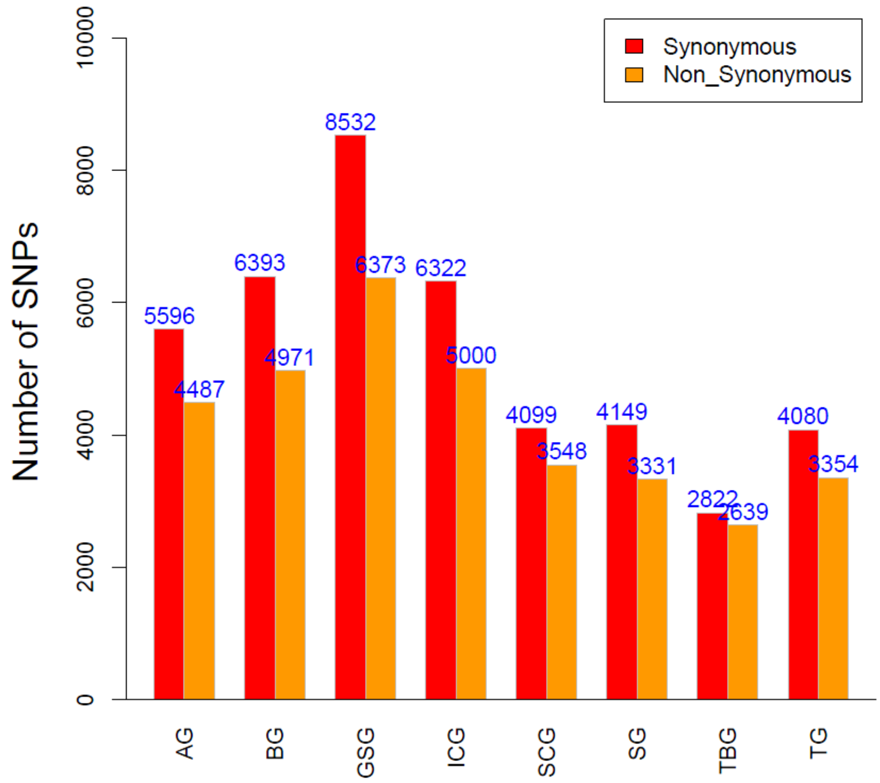

Supplementary Fig. S2 The number of synonymous and non-synonymous mutations in each goat breed. Angora (AG), Boer (BG), Guizhou Small (GSG), Inner Mongolia Cashmere (ICG), Shaanbei Cashmere (SCG), Saanen (SG), Taihang Black (TBG), Tibetan (TG).

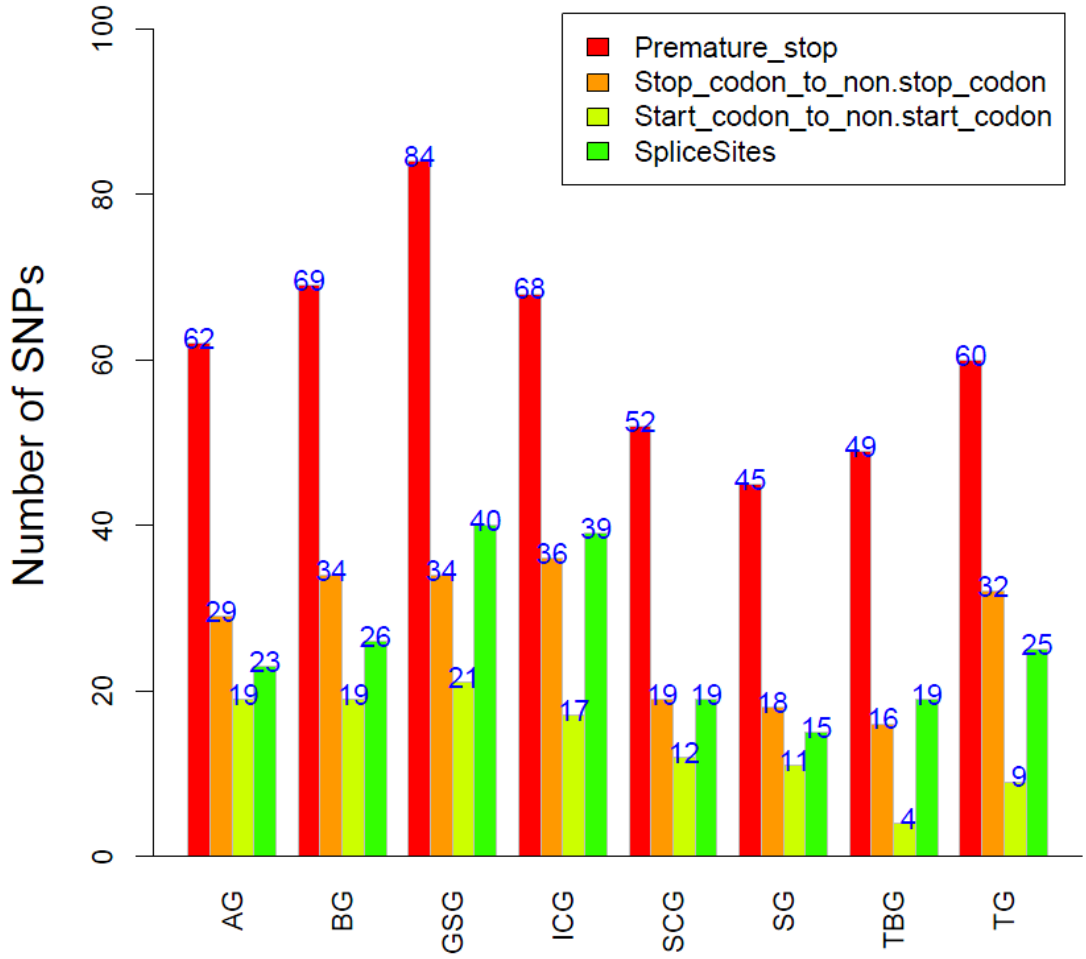

Supplementary Fig. S3 The number of SNPs with large effects (premature stop, stop codon to nonstop codon, start codon to nonstart codon, and splicing sites) in each goat breed. Angora (AG), Boer (BG), Guizhou Small (GSG), Inner Mongolia Cashmere (ICG), Shaanbei Cashmere (SCG), Saanen (SG), Taihang Black (TBG), Tibetan (TG).

Supplementary Table S1. Pooled samples information for resequencing data from goats. Goat DNA pools represent eight separate breeds respectively. The number of individuals from each breed (n) and the number of males and females, the average sequence and assembly coverage per pool are indicated.

Breed	No. of samples	Sequence coverage (x)	Phenotype/Feature
Taihang Black	(M=10, F=11)	9.35	Meat, black coated
Tibetan goat	(F=25)	13.95	Highland adaption
Inner Mongolia Cashmere	(M=8, F=20)	9.67	Fiber (cashmere)
Shaanbei Cashmere	(M=9, F=20)	9.13	Fiber (cashmere)
Angora	(M=10, F=10)	10.65	Fiber (mohair)
Saanen	(M=6, F=15)	10.86	Dairy
Boer	(M=7, F=15)	13.95	Meat, higher body weight
Guizhou Small	(M=3, F=18)	10.00	Meat, lower body weight

Supplementary Table S2. The number of candidate regions and genes identified by ZH_p and di in different breeds.

Breed	ZH_p	di	Overlapped regions	Overlapped genes
Taihang Black	48	54	6	6
Guizhou Small	49	56	4	4
Inner Mongolia Cashmere	40	37	5	5
Tibetan goat	49	53	7	6
Total	182	200	22	21
